# Dental health awareness in patients feeling sad or hopeless- an estimate from youth risk behavior surveillance survey

**DOI:** 10.1192/j.eurpsy.2021.894

**Published:** 2021-08-13

**Authors:** Y.C. Hsieh, B. Chauhan, J. Patel, P. Soni, G. Tyagi

**Affiliations:** 1 School Of Public Health, Icahn School of Medicine at Mount Sinai, New York City, United States of America; 2 Public Health, Long Island University School of Public Health, New York City, United States of America; 3 Dentistry, Rutgers School of Dental Medicine, Newark, United States of America

**Keywords:** Depression, Youth Risk Behavior Survey, Suicide, Dental health

## Abstract

**Introduction:**

Research has found that low mood including sadness and hopelessness is an important factor for decreased awareness in one’s oral health and lowered frequency of visit to the dental office, this relationship is not well studied in national representative samples. Poor mental wellbeing can lead to poor oral health.

**Objectives:**

Aim is to examine the relationship between feeling sad or hopeless and awareness for dental care.

**Methods:**

Data were obtained from the Youth Risk Behavior Surveillance Survey (YRBS-CDC), USA, for years 2009-2017. All ages from 12 to 18 years, feeling sad or hopeless and dental visits were identified. Univariable relationship between feeling sad or hopeless (>2 weeks in the past year) and dental office visits for all dental care (during the past 12 months, 12-24 months, >24 months, and never) was evaluated using chi-square test.

**Results:**

Out of a total of 53,098 youths, 30.5% of youths were feeling sad or hopeless. Within the youths feeling sad or hopeless, the prevalence of youth who never received dental care was higher at 37.1% in comparison to youths who received dental care >24 months ago 36.4%,12-24 months 33.7%, and visited the dental office in the past 12 months 28.9%. (p<0.0001). In youths who had dental care in the last 12 months, the prevalence of sadness or hopelessness was lower at 65% vs 70%, while it was higher in youths who had never had dental care at 3.3% vs 1.7%.

**Conclusions:**

Further research is warranted to evaluate reduced oral health care awareness among participants feeling sad or hopeless.

